# Mindfulness based stress reduction for medical students: optimising student satisfaction and engagement

**DOI:** 10.1186/s12909-016-0728-8

**Published:** 2016-08-18

**Authors:** Declan Aherne, Katie Farrant, Louise Hickey, Emma Hickey, Lisa McGrath, Deirdre McGrath

**Affiliations:** 1 Counselling Department, University of Limerick, Limerick, Ireland; 2 Graduate Entry Medical School, University of Limerick, Castletroy, Limerick, Ireland; 3 Trinity College Dublin, Dublin, Ireland

**Keywords:** Mindfulness, Stress reduction, Doctor burnout, Academic stress in medical students, Self-care, Student satisfaction, Medical undergraduate

## Abstract

**Background:**

Medical practitioners and students are at increased risk of a number of personal and psychological problems. Stress and anxiety due to work-load and study requirements are common and self-care methods are important in maintaining well-being. The current study examines perceptions of and satisfaction ratings with a mindfulness based stress reduction (MBSR) programme for 1^st^ year (compulsory) and 2^nd^ year (optional) Graduate Entry Medical School students.

**Methods:**

A mixed method pre and post study of Year 1 (*n* = 140) and Year 2 (*n* = 88) medical students completing a 7 week MBSR course compared student satisfaction ratings. Thematic analysis of feedback from the students on their perception of the course was also carried out.

**Results:**

Year 1 students (compulsory course) were less satisfied with content and learning outcomes than Year 2 students (optional course) (*p* < .0005). Thematic analysis of year 1 student feedback identified themes including great concept, poorly executed; and less discussion, more practice. Year 2 themes included session environment and satisfaction with tutors.

**Conclusions:**

The MBSR course was associated with high levels of satisfaction and positive feedback when delivered on an optional basis. Catering for the individual needs of the participant and promoting a safe environment are core elements of a successful self-care programme.

**Electronic supplementary material:**

The online version of this article (doi:10.1186/s12909-016-0728-8) contains supplementary material, which is available to authorized users.

## Background

A large body of research exists which suggests that medical practitioners and medical students may be at increased risk of a number of personal and psychological problems. Dealing with the suffering of others can have a negative impact on health professionals, resulting in psychological illnesses, suicide, depression, anxiety and alcoholism [1, 2]. Suicide rates have been reported to be higher among doctors than among the general population [3, 4] while life satisfaction has been found to be lower among this cohort than among an educationally and age-matched sample of the general population [5]. The mental health, wellbeing, and life satisfaction of doctors have been found to be negatively impacted by high psychological demands, low job control, low levels of support from co-workers and supervisors [6–8], and they are influenced by individual factors such as relationship status, age, and personality traits [5]. The overwhelming demands of their work prevent them from paying adequate attention to their own wellness [9, 10]. These demands can often result in burnout, which can lead to decreased job satisfaction, and an increased likelihood to make medical errors [1, 2, 9, 11, 12].

Medical students have been shown to have high levels of stress, depression [13–15], mental distress, burnout [14], as well as low life-satisfaction [16]. Some studies have suggested that these psychological difficulties are higher among medical students than among students of other courses [17] or a matched cohort of the general population [18], while other studies have found similar stress levels in engineering students [19] and psychology students [16]. Stress can negatively affect the emotional, psychological and social well-being of medical students [20], leading to depression, suicidal ideation, burnout, substance abuse, and other personal and professional problems [11, 21–23].

Academic stress, in addition to personal stressors, can lead to higher levels of depressive symptoms in medical students [24], as well as reducing overall mental well-being [22]. Stress experienced at medical school has also been shown to predict problems experienced as qualified doctors [25]. Maladaptive coping styles and stress in medical students occur due to high workload, low time management skills, unrealistic expectations, low confidence in one's own ability, and high levels of competition rather than co-operation in medical schools [26]. These maladaptive coping styles correlate with psychological difficulties such as stress, anxiety, and depression [17]. The personal and psychological difficulties experienced by many medical doctors and students can negatively impact on patient care [1, 2, 9, 16, 27]; reduce the ability to empathise with patients [28], and decrease the ability to connect with patients [29].

These issues highlight the importance of self-care among medical students and doctors. Finding time to take care of these personal difficulties can lead to increased wellbeing and career satisfaction, reduced stress, and the likelihood of making medical errors [30], while increased self-compassion can strengthen client relationships, increase well-being, and reduce empathetic distress, fatigue and burnout [31]. However, doctors have been found to be less likely to seek medical help, and may minimise any psychological issues they experience [1, 2, 10]. This may be due to fears that being a patient is a sign of weakness that makes them unfit to work as a doctor, while there are also issues around confidentiality if they were to be treated by a colleague [1, 2, 32]. Researchers and therapists have suggested that a successful self-care course for medical students, as well as focusing on reducing stress and increasing well-being, should aim to target academic as well as personal stressors [24], promote positive coping strategies [33], and include elements of reflective practice [34]. A study by Krasner et al. [35] found that physicians who participated in a Continuing Medical Education (CME) program on mindful communication demonstrated increased levels of well-being and an enhancement in personal attributes associated with a more client-centred clinical care. Findings from a more recent study by West et al. [36] also support these results. They staged an intervention which involved 19 biweekly facilitated physician discussion groups which included elements of mindfulness, reflection, shared experience, and small-group learning for nine months. It was found that this improved meaning and engagement in work and reduced depersonalisation, with sustained results at 12 month follow-up. Wald et al. [37] report the significance of reflective writing as a ‘metaphorical resiliency workout’, aiding in the development of mindful reflective practice to promote resilience and to enrich learners’ well-being, as part of overall Professional Identity Formation (PIF). As evidenced above, much of the research on self-care courses for medical students and physicians has features of Mindfulness-Based Stress Reduction (MBSR) [38] which can be adapted to suit the needs and schedules of medical students.

An abbreviated version of MBSR has been shown to be effective for caregivers [39], nurses, midwives [40], support staff working in intellectual disability [41], trainee mental health therapists [42], social work students [43], and other college students [44]. There is a growing body of research demonstrating the positive effects of MBSR on the physical and mental health of medical students and health-care providers [9, 45–47], leading to reductions in stress, anxiety, psychological distress [23], self-doubt, and concentration loss [48], along with increased self-compassion [29, 39], self-awareness [49], empathy [23, 29], and positive affect [50]. MBSR has also been demonstrated to improve selective attention, sustained attention, conscious perception, visual working memory [51, 52], and increased knowledge retention [53], along with other cognitive, emotional, and spiritual benefits [54].

Programs using Mindfulness based meditation in medical schools have also become more popular e.g. the University of Massachusetts Medical School have a Centre for Mindfulness in Medicine, Health Care and Society and offer an MBSR in Mind-Body Medicine: 7-Day Residential Professional Training as well as other mindfulness based training courses. A study by Saunders et al. [55] found that first year students at Georgetown University School of Medicine who participated in the 11-week long Mind-Body Skills (MBS) reported benefits such as self-discovery, learning, stress relief, and medical education. Other advantages included making the stress of medical school more manageable; presenting the opportunity for self-care; improving a sense of community and social support among students; and promoting an openness towards MBS. Participants involved in this study chose the MBS program as part of an elective course, for which no course credits were given, and were therefore highly motivated to participate and learn from the program from the outset.

A number of considerations need to be taken into account when designing an MBSR course for medical students. The students' perceptions of the need for the course, along with motivation to engage in the course may affect the benefits they gain [49]. College students have been found to experience difficulties including cognitive and physical challenges, time constraints, motivation, and disbelief in the efficacy of meditation [54]. Not being open to the experience of mindfulness can result in participants being critical of the technique or themselves, hindering their participation in the practice [56], while the degree and depth of engagement with mindfulness can affect psychological health outcomes [57]. If students are not open to the experience and do not perceive the benefits, they are less likely to benefit from the programme.

MBSR programmes for medical schools are often abbreviated due to time constraints. A systematic review of both the standard and adapted MBSR programmes did not find any correlation between mean effect size and the number of course hours [58]. This suggests that, although most empirical support for MBSR is based on studies of the standard eight classes, abbreviated versions may be equally effective and may be more suitable where time and resource constraints occur.

The Health Enhancement Program (HEP) at Monash University comprises mindfulness and ESSENCE lifestyle programs, is experientially-based, and integrates with biomedical sciences, clinical skills and assessment. A cohort study by Hassed, de Lisle, Sullivan and Pier [59] performed on the 2006 first-year intake measured effects of the HEP on various markers of wellbeing. A total of 148 of an eligible 270 students returned data and improved student wellbeing was noted on all measures and reached statistical significance for depression and hostility subscales and the General Severity Index (GSI) of the Symptom Checklist 90 (SCL-90), but not the anxiety subscale. Statistically significant results were also found for the psychological domain but not the physical domain of the World Health Organisation Quality of Life (WHOQOL) assessment. This study was the first to demonstrate an overall improvement in medical student wellbeing during the pre-exam period suggesting that the common decline in wellbeing is avoidable.

The current study examines perceptions of and satisfaction ratings with a mindfulness based stress reduction programme for 1^st^ year and 2^nd^ year Graduate Entry Medical School students during their academic year 2013 / 14. In particular this study compares the use of a compulsory versus an optional approach to mindfulness course participation on student perceptions of and satisfaction with the course. Qualitative and quantitative analysis of student feedback in relation to the course and satisfaction with the course were carried out to inform future programme delivery of self-care courses.

## Methods

Both 1^st^ and 2^nd^ year MBSR courses are facilitated by freelance psychotherapists who are trained in mindfulness, and are under the supervision of a Clinical Psychologist with 25 years of mindfulness experience. They are employed by the University of Limerick Graduate Entry Medical School (GEMS) to deliver the course which is based on Jon Kabat Zinn's Mindfulness Based Stress Reduction course [37] and adapted to meet the needs of the medical students . This involved maintaining the original content of the MBSR course but condensing it in terms of time (reduced from 8 weeks to 7 weeks; 1 to 2 h per week).

Prior to commencing the course, all students were given a one hour presentation introducing them to the principles of and evidence for mindfulness as well as its relevance for medical students and how it was integrated within the medical degree programme and clinical practice. The course contributed 25 % towards the Professional Competencies module for medical students, with the remaining components including psychology, sociology and ethics. The course tutors were experienced mindful practitioners and psychotherapists who had at least two years experience working with medical students. Assessment of the module was on a pass / fail basis. For this component students pass the module having attended and participated, including doing homework, which consisted of formal and informal practice. Logbooks of their practice were submitted at the end of the course. The course itself consisted of seven weeks of 1 h / 2 h teaching and practice. The emphasis in these classes was on the experiential aspect of mindfulness practice. Time was also given to discuss the principles and the applications for students to their daily lives and their medical careers. The course was clearly structured to provided training / exposure to mindfulness self-enquiry and practices including mindfulness of the breath, sitting meditation, choiceless awareness, body-scan, eating mindfully and walking mindfully. There was also some flexibility from session to session, depending on what issues arose for participants from week to week in their class. The course content for both first and second years was exaclty the same albeit the duration of the sessions for second years was longer.

### Participants: 1^st^ year medical students

A purposeful sample of 140 participants (Male = 64 (45 %); Female = 71 (53 %), 5 participants did not identify with a particular gender) were recruited from the University of Limerick 1^st^ year medical student community. This comprised all first year students as the course is compulsory for this year cohort. Participants ages ranges from 20 to 43 years (*M*_*age*_ = 24.4 years, *SD* = 3.4). Participants were recruited through their involvement in a compulsory one hour per week, seven week Introduction to Mindfulness Based Stress Reduction program (MBSR) as part of the Graduate Entry Medical School (GEMS) first year syllabus (Intervention group).

### Participants: 2^nd^ year medical students

A purposeful sample of 88 (Male = 39 (45 %), Female = 48 (55 %) 1 participant did not identify with a particular gender) students comprising all second year students who untertook the course was recruited from the University of Limerick 2^nd^ year medical student community. Their ages ranged from 22 to 37 years (*M*_*age*_ = 26.2 years, *SD* = 3.1). Participants were recruited through their involvement in an optional two hour per week, seven week Mindfulness Based Stress Reduction program (MBSR) as part of the GEMS second year syllabus (Intervention group: *n* = 88). Their alternative option was to complete a written assignment on self-care. 23 students did not participate in the formal programme and availed of this option instead. These students were therefore excluded from the current study, and no feedback was obtained as groups were not completing comparable assignments.

Inclusion criteria for all participants was English language proficiency, as this was the primary language of the researchers and all questionnaires utilised in the study were designed for English speakers.

#### Materials

All participants were given an open-ended qualitative feedback questionnaire with a satisfaction rating scale (see Additional file 1: Figure S1: Qualitative Feedback Questionnaire**)** as well as a demographics questionnaire requesting gender, age, university course, and whether they had any previous experience of mindfulness. All questionnaires were coded with student numbers and initials and were anonymous to researchers.

#### Procedure

Participants were approached at the beginning of their mindfulness course and asked to take part in the study. An information sheet and informed consent forms were distributed at this point. The research questionnaires were given to those who agreed to participate, once they had completed their course.

#### Analysis

A t-test analysis was used to compare the quantitative data of satisfaction ratings for 1^st^ and 2^nd^ years using SPSS Version 21. A 5 % level of significance was used for all statistical tests. Qualitative feedback was analysed using thematic analysis [60]. Thematic analysis is the most common form of analysis in qualitative research. It pinpoints, examines, and records patterns (or "themes") within data and the themes become the categories for analysis. Thematic analysis is performed through a process of coding to create established, meaningful patterns.

## Results

### Quantitative results

Comparing 1^st^ and 2^nd^ year medical students’ satisfaction questionnaire results:

140 of Year 1 students and 88 of year 2 students completed the questionnaires after the final mindfulness session, with no significant differences between Year 1 and Year 2 in numbers of sessions attended [t(187) = .48, *p* = .63]. Satisfaction ratings on all five items assessed for both 1^st^ and 2^nd^ years were above or at / close to the mid-mark of ‘3’ on the five point likert-scale. There were no significant differences between Year 1 and Year 2 in terms of levels of satisfaction with trainer [t (191) = -1.17, *p* = .24]. Significant differences were found between Year 1 and Year 2 on satisfaction with course content [t(191) = -6.2, *p* < .0005], satisfaction with learning outcomes [t(142) = -4.5, *p* < .0005], overall satisfaction [t(190) = -5.18, *p* < .0005], and whether they would recommend to others [t(191) = -5.16, *p* < .0005], with the second year students scoring higher on these items, indicating a greater satisfaction with the course. Table 1 outlines the mean and standard deviation of each item on the feedback sheets.

**Table 1:** Mean scores on satisfaction questionnaires for Year 1 and Year 2

	Year 1 (*n* = 140)		Year 2 (*n* = 88)		t-test
Item	Mean	Std. Deviation	Mean	Std. Deviation	*p*
No. Sessions attended out of seven	6.49	.61	6.44	.55	.63
On a scale of 1 to 5:					
Satisfaction with Content	3.04	.89	3.86	.91	<.0005
Satisfaction with Trainer	4.08	.81	4.23	.88	.24
Satisfaction with Learning Outcomes	3.01	.89	3.65	.999	<.0005
Overall Satisfaction	2.98	.96	3.72	.94	<.0005
Recommend to others	2.87	1.09	3.7	1.07	<.0005

These results would indicate that all participants were reasonably to well satisfied with all aspects of their course, with 2^nd^ years demonstrating a significantly higher level of satisfaction than first years on four of the five factors measured.

### Qualitative results

At the end of the satisfaction questionnaire, students were given the option of making "further comments/suggestions/ feedback". Of the Year 1 students who completed the satisfaction questionnaires, 88 (62.8 %) provided comments, while 35 (39.8 %) of the Year 2 respondents gave additional comments. A thematic analysis was conducted on the responses. The qualitative data for the Year 1 and Year 2 students were analysed separately to allow for comparison.

The comments and feedback from the satisfaction questionnaires was transferred into a table in a Word document (one line per participant). Line by line coding was carried out, staying as close to the data as possible. Not all of these codes were used when generating the themes, as some were only mentioned by one participant, or were perceived as having little relevance to either the subject or the themes. Ideas of themes began to become clear to the researchers during transcription and coding where there were numerous comments of a similar nature (e.g. "Satisfaction with Tutors"). The final identified themes can be seen in Table 2.

**Table 2:** Year 1 and Year 2 Themes in order of most common

Year 1 Themes	Year 2 Themes
Theme 1: Great concept, poorly executed Theme 2: Satisfaction with MBSR course Theme 3: Cater for Individual Preferences Theme 4: Less discussion, more practice	Theme 1: Cater for individual preferences Theme 2: Benefits of MBSR course Theme 3: Satisfaction with Tutors Theme 4: Session environment

### Year 1 Themes

#### Theme 1: Great concept, poorly executed

Although many of the first year students expressed satisfaction with the course, the most common theme suggests that students did not feel the delivery of the course was satisfactory. As one participant succinctly stated:

*I think the concept of the module is great, but the delivery is less than relevant*

In particular, the students referred to the "bad timing" of the module, which was mentioned 28 times and was the most commonly reoccurring issue. This refers to a number of issues, including the time of day the sessions were on:

*The timing of the sessions is not good - after academic activities (e.g. 4:15 pm) would be a better time.*

*Winding down in the middle of the day is not ideal for a med student*

The course was scheduled on the students' PBL (problem-based learning) day, which is a busy and stressful day for the students:

*The timing of the module scheduled on a Tuesday, close to our PBL which was quite stressful in itself. I think due to this, participation and enjoyment perhaps waned.*

*I feel that Tuesday morning was our most stressful time and I would have been more receptive to the course had it been on another day.*

Partly because of these scheduling problems, the mindfulness sessions were occasionally seen as an additional source of stress to the students, interfering with their other course work:

*Being forced into assessing your stress while stressed is quite stressful*

*I don't get stressed often so it was irritating having to relax even more through meditation when I could've been getting work done*

This theme suggests that, while many of the students may have been open to mindfulness practice however the way in which this particular course was delivered reduced students' ability to engage fully.

#### Theme 2: Satisfaction with MBSR course

Another common theme was the students' perceptions about the benefits of mindfulness. Some of the students felt that mindfulness is a useful skill to have:

*Great idea, great intentions - learnt a lot about control of stress.*

*The idea is great, it's a fantastic ability to be able to practice mindfulness*

The perceived benefits of mindfulness include gaining an awareness of your own stress and having the skills to reduce this.

*I do think the course is good and helped me realise how stressed I really am.*

*Mindfulness is definitely a good practice for people to manage stress in my opinion*

Others felt that mindfulness is only useful to those who feel stressed, and that it has no benefits for those who have their own coping mechanisms:

*Recommend if one is struggling. If one is genuinely happy this is not needed*

*If you have no capacity to relax, then this is very relevant - but if you can then this was almost stressful to keep on top of*

While many of the participants enjoyed the course, quite a few found it difficult, unsatisfying, and frustrating:

*I loved the mindfulness sessions, great to relax*

*Didn't like mindfulness. Pains me to sit still.*

*Could not relate to the content and the practices. Felt too "hippie". Could not focus during meditation*

#### Theme 3: Cater for individual preferences

Another common theme was the importance of recognising individual differences in coping styles etc. and the need to adapt courses to suit students' preferences. A number of individuals noted that mindfulness is "Not suitable for everyone", while a common issue which arose was the desire for a variety of exercises, including mindfulness exercises, physical exercises, and other activities for reducing stress:

*I would suggest incorporating other methods of stress-relief instead of focusing all 7 weeks on mindfulness training.*

*Forcing people to try to relax in a particular way is ridiculous given the different methods people use to relax. Weeks would be much better spent sampling a number of different relaxation methods.*

The mandatory nature of the course was a source of particular discontent among the students, with many feeling that this is "counterproductive" and that a willingness to engage in mindfulness is essential - it cannot be forced:

*Would only recommend if the person wanted to attend, being obligatory to attend meant that even if not open to the practice of mindfulness you still had to attend & I don't think you would get as much out if it as you would if it had it been optional.*

Being "forced" into practicing mindfulness was seen to have a negative effect on some individuals who then disrupted their fellow classmates:

*Some students in the group make it very difficult to relax, so I think there should be an alternative module for these people as it effects the overall experience.*

*Make it optional - does not suit some personalities, may ruin it for others.*

This theme strongly suggests that medical students would prefer a stress reduction course which is better suited to their specific needs as a group and as individuals.

#### Theme 4: Less discussion, more practice

The final theme refers to the amount of time spent discussing stress-reduction techniques rather than putting those techniques into practice:

*The course would be better if we actually spent time practicing stress relieving techniques instead of simply discussing them.*

*Less time talking about stress, more time doing activities to reduce stress.*

Others simply stated preferences for different techniques, activities, and skills which they had found useful, suggesting that these are incorporated more on future courses:

*Meditation isn't for everyone, alternate it with yoga and other stress relievers*

*I would have liked to do more practical exercises e.g. yoga, body scan and learnt more practical ways to practice mindfulness as opposed to how stressed I feel/what stress is.*

Summary of qualitative data for 1^st^ years.

Four distinct themes emerged from this data, all of which had some negative connotations. The issue of the compulsory nature of the course was expressed in terms of students feeling that individual preferences of self-care approaches were not catered for. Poor scheduling of classes, poor design of sessions and a dissatisfaction with the concept of mindfulness were further sources of disgruntlement for this cohort.

### Year 2 Themes

#### Theme 1: Cater for individual preferences

This theme also arose among the first year students, and is similar in its content. Many of the students expressed their own preferences for different activities:

*I would prefer a more grounded program, less concerned with metaphysical concepts (often offered in the body scan)*

*Introduce mindful walking, yoga, and S.T.O.P. as early as possible.*

Others discussed the necessity of being willing to engage in the course:

*My recommendation depends on the attitude of the student towards the program – need to be open to it to get the most out of it.*

Unlike the first year module, the second year module is optional. Therefore, the issue of mindfulness being "mandatory" or "forced" did not arise. Another difference between the first and second year students was that no reference to being disrupted by unwilling classmates was mentioned by the second year students.

#### Theme 2: Benefits of MBSR course

This is related to the first year theme of "Satisfaction with MBSR course", although the content differs in some ways. The second year students' expressed positive views, discussing ways in which the course has helped them, how much they have enjoyed it, and comparing it with their experiences in first year.

*The course has helped me beyond these 7 weeks & I am grateful for the reference.*

*Brilliant, really helped during a hard breakup*

It fostered understanding and learning:

*Great experience really learnt a lot and took a lot from it.*

*Great craic while learning about myself and others.*

A number of participants compared this year's course to the one they completed in first year:

*Overall, good experience. I learned/understood more about mindfulness this year vs. last.*

*Found module very good. Would participate again. Better content than last year.*

One participant reported finding it difficult to engage with the course:

*I sometimes wondered how mindfulness differed from other coping mechanisms, sometimes it was difficult to see how useful/relevant the practice was in daily life*

#### Theme 3: Satisfaction with tutors

While comments specifically about the tutors did not occur very often among the first year feedback, this was one of the most common themes amongst the second years. The tutors were described as "brilliant", "a fantastic tutor", "an amazing instructor" etc. Related to Theme 1 (cater to individual preferences), one of the reasons the tutors received such positive reviews was because they tailored the course to suit the students rather than expecting the students to change to suit the course:

*I really liked that our tutors listened and gave us what we wanted to do!! They tailored the 7 weeks for us!! Rather than us to the course.*

#### Theme 4: Session environment

The fourth most common theme among the second years includes the nature of the environment within the mindfulness sessions and how this can impact either positively or negatively on students' experiences. Having a safe environment in which to de-stress was seen as important:

*My program doesn’t allow us to take time out for ourselves, so it was beneficial to do so in a safe environment.*

Being able to trust those in your group was also seen as beneficial:

*Absolutely brilliant. Really helped me and I felt as a group there was a great bond and trust between us.*

One negative aspect which students reported was a sense of judgement in some of the sessions. This seems to have arisen from a particular incident which left some students feeling they had been misunderstood:

*I found a sense of judgement pervaded sessions.*

*At times I felt negative judgement from instructors after misinterpreting my comments.*

This only featured in the comments of 2 participants. Overall, the participants felt that the session environment was positive, and contributed to their enjoyment of the course.

Summary of qualitative results for 2^nd^ years:

In contrast to the tone of first years, the second year’s themes were of a generally positive nature. Students reported the perceived benefits of mindfulness and their satisfaction with their tutors and the class environment. Second years did make mention of the need to cater for individual preferences, similar to first years. As the course was optional for second years, this factor cannot be considered as being due to students feeling compelled to do it and so we may need to re-visit our analysis of this factor in our conclusions.

## Discussion

This study sought to examine the perceptions of and satisfaction ratings for Year 1 and Year 2 graduate entry medical students and to determine whether an optional versus compulsory approach to this type of programme might have an impact on perception and on satisfaction levels.

Quantitative findings indicated that Year 2 students had higher levels of satisfaction with course content and learning outcomes. While the optional nature of the course in Year 2 may have been a factor in heightening satisfaction levels, it is possible that other factors such as 1) having previously been exposed to mindfulness in Year 1, 2) a longer course than that of Year 1 students and 3) unlike their Year 1 counterparts having had clinical placement experience may also have had an impact.

The qualitative results confirm that the second year students were more satisfied with their course, felt more positively towards it, and engaged with it to a greater extent than the first year students. This again may reflect a greater understanding by the second year students of the relevance and broader application of mindfulness with a longer period of engagement with the course. It may also reflect deeper perception by students who have had the opportunity to experience the relevance of the course to clinical practice having undergone clinical placement exposure. The importance of clinical relevance and duration of a course is strengthend by the work of West et al demonstrated improved meaning and engagement with work and reduced depersonalization amongst physicians in practice with a prolonged mindfulness based programme [36].

One might argue that the course employed in this current study was weakened by the fact that it was customised for medical students. However, West et al provided a customised programme of mindfulness with the successful outcome demonstrating that it is possible to remain faithful to the philisophy of mindfulness while being flexible with the format in which is delivered.

One of the most frequently cited difficulties reported by the first years, which seems to have greatly impacted on their ability to engage with the course, was the inconvenient positioning of the course on what they described as their most demanding and stressful day. It is possible that stressful work demands were in conflict with taking time out for self-care. In feedback from students, it was noted that the classes were also held on a PBL day for the second year cohort but they did not identify workload as a major concern. As discussed, openness to the experience of mindfulness is considered to be essential if one is to reap the benefits [56], and this may have reduced the effectiveness of the course for the first year students. It may therefore be worth considering changing the course to another day of the week, if possible, or scheduling evening classes instead of morning classes. The possibility that there may never be a ‘right time’ within medical training for self-care also needs to be acknowledged, highlighting a more systemic problem within medical education more generally.

Many 1^st^ year students felt that, if you are being "forced" to take part in mindfulness, then you cannot truly engage with the practice, which requires openness to the experience, a potential explanation of reduced satisfaction with the course compared to 2^nd^ year students. Indeed the very ethos of mindfulness recognises and values the importance of individual choice and responsibility. Perhaps, if there was some element of choice, the students would feel more empowered and take a greater interest in the course, as with the second year students who reported that their tutors listened to their requests and catered the course to their needs and preferences. These findings are congruent with those of Dobkin & Hutchinson [49] mentioned previously. Careful consideration needs to be given to planning the content and delivery of this type of programme to incoming students, where a balance is reached between recognising individual preferences and prioritising the importance of doctor self-care as a part of medical training. In this instance, prospective students would have been informed through the course syllabus prior to entry that this course was an integral part of the medical programme they were choosing to come on.

Another issue which emerged was that students often reported a poor understanding of the purpose of mindfulness but also felt that spending too much time discussing this in their sessions instead of implementing the techniques was a waste of time and counterproductive. It is possible that the group preferred to learn through experiencing the techniques rather than speaking about them, but further investigation is needed on this. Going forward, a solution may be to run a workshop outlining the effectiveness of mindfulness in stress reduction prior to undertaking the course. Having a prior knowledge of the benefits of MBSR, the second year students may have a greater motivation to engage with a stress reduction course than the first year students, who viewed the course as an addition to their workload. While the approach to facilitation was possibly more instructive for first year students a less didactic approach to facilitation in the second year fostered a more learner-centred approach, promoting a more reflective culture (“reflection before action”) in relation to relationship-centred education and effective mentoring. It is worth noting that without the foundation work achieved in 1^st^. year, that the gains that were made in 2^nd^ year might not have been possible.

The qualitative data in this study was both rich and informative but was limited due to the questionnaire format. Considering the complex nature of mindfulness, using focus groups or a small sample of individual interviews may provide a more detailed qualitative evaluation of the effectiveness of this type of programme and of student perceptions in relation to it. In order to discern the long-term benefits of the MBSR course studies need to be conducted following qualification to measure negative and positive outcomes, and ongoing use of mindfulness practices.

## Conclusion

Mindfulness based stress reduction programmes are associated with high levels of satisfaction in particular when delivered on an optional basis. These satisfaction levels appear to increase over time and with increased understanding of the relevance of the course as students progress through their medical degree programme and clinical placement opportunities. Further factors to be considered when delivering self-care for medical students include recognition of scheduling and time-tabling demands for students, students understanding of the concept of mindfulness and why it is relevant for medical students, students’ openness to learning, and students’ incoming levels of stress. Some inconsistencies identified in this study may reflect an inherent discomfort and resultant resistance to self-care among medical students, which is manifest in various objections to doing the module until such time as they acquire a better understanding of its nature and relevance. Satisfaction with such courses may be further ameliorated by creating real life clinical placement experiences so that students may appreciate the true role of stress reduction measures. More detailed qualitative studies in this area are clearly needed.

Additional file 2 (Practice Points) provides a list of key practice points from this study which may prove useful to the reader.

## Additional files

Additional file 1: Figure S1. Qualitative Feedback Questionnaire. Open ended feedback questionnaire. (DOCX 69 kb) Additional file 2: Practice Points. List of practice points relevant to the study. (DOCX 51 kb)

## Abbreviations

GEMS: Graduate entry medical school
GSI: General severity index
HEP: Health enhancement programme
MBSR: Mindfulness based stress reduction
SCL-90: Symptom checklist 90

## References

[1] Brown C (2008). Doctors' health matters - learning to care for yourself. J Holistic Healthc.

[2] Knight R (2011). The doctor, the patient and compassion. J Holistic Healthc.

[3] Rosta J, Aasland OG. Changes in the lifetime prevalence of suicidal feelings and thoughts among Norwegian doctors from 2000-2010: a longitudinal study based on national samples. BMC Psychiatry. 2013; doi: 10.1186/1471-244X-13-322 10.1186/1471-244X-13-322 PMC4219507 24286517

[4] Stack S (2004). Suicide risk among physicians: A multivariate analysis. Arch Suicide Res.

[5] Tyssen R, Hem E, Gude T, Gronvold NT, Ekeberg O, Vaglum P (2009). Lower life satisfaction in physicians compared with a general population sample. Soc Psychiatry Psychiatr Epidemiol.

[6] Escriba-Aguir V, Perez-Hoyos S (2007). Psychological well-being and psychosocial work environment characteristics among emergency medical and nursing staff. Stress Health.

[7] Imran N, Haider II, Iqtader S, Bhatti MR (2011). Unhappy doctors in Pakistan: What are the causes and what can be done?. Pak J Med Sci.

[8] Jones MC, Wells M, Gao C, Cassidy B, Davie J (2013). Work stress and well-being in oncology settings: a multidisciplinary study of health care professionals. Psycho-Oncology.

[9] Irving JA, Dobkin PL, Park J (2009). Cultivating mindfulness in health care professionals: A review of empirical studies of mindfulness-based stress reduction (MBSR). Complement Ther Clin Pract.

[10] Wallace JE, Lemaire J (2009). Physician well being and quality of patient care: An exploratory study of the missing link. Psychol Health Med.

[11] Prins JT, Hoekstra-Weebers JEHM, Gazendam-Donofrio SM, Dillingh GS, Bakker AB, Huisman M (2010). Burnout and engagement among resident doctors in the Netherlands: a national study. Med Educ.

[12] Romani M, Ashkar K (2014). Burnout among physicians. Libyan J Med.

[13] Haldorsen H, Bak NH, Dissing A, Petersson B (2014). Stress and symptoms of depression among medical students at the University of Copenhagen. Scand J Public Health.

[14] Kittu D, Patil R (2013). Study of association of psychological stress and depression among undergraduate medical students in Pondicherry. Nat J Commun Med.

[15] Yusoff MSB, Rahim AFA, Baba AA, Ismail SB, Pa MNM, Esa AR (2013). The impact of medical education on psychological health of students: A cohort study. Psychol Health Med.

[16] de Vibe M, Solhaug I, Tyssen R, Friborg O, Rosenvinge JH, Sorlie T (2013). Mindfulness training for stress management: a randomised controlled study of medical and psychology students. BMC Med Educ.

[17] Wong JGWS, Patil NG, Cheung VW, Chan LC, Mak FL (2015). Cultivating psychological well-being in Hong Kong's future doctors. Med Teach.

[18] Biro E, Balajti I, Adany R, Kosa K (2010). Determinants of mental well-being in medical students. Soc Psychiatry Psychiatr Epidemiol.

[19] Behere SP, Yadav R, Behere PB (2011). A comparative study of stress among students of medicine, engineering, and nursing. Ind J Psychol Med.

[20] Michalec B, Keyes CLM (2013). A multidimensional perspective of the mental health of preclinical medical students. Psychol Health Med.

[21] Mavor MI, McNeill KG, Anderson K, Kerr A, O'Reilly E, Platow MJ (2014). Beyond prevalence to process: the role of self and identity in medical student well-being. Med Educ.

[22] Nandi M, Hazra A, Sarkar S, Mondal R, Ghosal MK (2012). Stress and its risk factors in medical students: an observational study from a medical college in India. Indian J Med Sci.

[23] Shapiro SL, Scwartz GE, Bonner G (1998). Effects of mindfulness-based stress reduction on medical and premedical students. J Behav Med.

[24] O'Reilly E, McNeill KG, Mavor KI, Anderson K (2014). Looking beyond personal stressors: An examination of how academic stressors contribute to depression in Australian graduate medical students. Teach Learn Med Int J.

[25] Tyssen R, Vaglum P, Gronvold NT, Ekeberg O (2001). Factors in medical school that predict postgraduate mental health problems in need of treatment. A nationwide and longitudinal study. Med Educ.

[26] Cherkil S, Gardens SG, Soman DK (2013). Coping styles and its association with sources of stress in undergraduate medical students. Ind J Psychol Med.

[27] Lucia-Casademunt AM, Ariza-Montes JA, Morales-Gutierrez AC (2013). Exploring European doctors' well-being by applying a neural network. HealthMED.

[28] West CP (2012). Empathy, distress and a new understanding of doctor professionalism. Med Educ.

[29] Bond A, Mason HF, Lemaster CM, Shaw SE, Mullin CS, Holick EA (2013). Embodied health: the effects of a mind-body course for medical students. Med Educ Online.

[30] Cedfeldt AS, Bower EA, English C, Grady-Weliky TA, Girard DE, Choi D (2010). Personal time off and residents' career satisfaction, attitudes and emotions. Med Educ.

[31] Boellinghaus I, Jones FW, Hutton J (2014). The role of mindfulness and loving-kindness meditation in cultivating self-compassion and other-focused concern in health care professionals. Mindfulness.

[32] Fältholm Y (2007). "Patients, not doctors, get sick": A study of fifteen Swedish physicians on long-term sick leave. Int J Qual Stud Health Well-being.

[33] An H, Chung S, Park J, Kim SY, Kim KM, Kim KS (2012). Novelty-seeking and avoidant coping strategies are associated with academic stress in Korean medical students. Psychiatry Res.

[34] Lutz G, Scheffer C, Edelhaeuser F, Tauschel D, Neumann M (2013). A reflective practice intervention for professional development, reduced stress and improved patient care - A qualitative developmental evaluation. Patient Educ Couns.

[35] Krasner MS, Epstein RM, Beckman H, Suchman AL, Chapman B, Mooney CJ, Quill TE (2009). Association of an educational program in mindful communication with burnout, empathy, and attitudes among primary care physicians. Jama.

[36] West CP, Dyrbye LN, Rabatin JT, Call TG, Davidson JH, Multari A (2014). Intervention to promote physician well-being, job satisfaction, and professionalism: a randomized clinical trial. JAMA Intern Med.

[37] Wald HS, Anthony D, Hutchinson TA, Liben S, Smilovitch M, Donato AA (2015). Professional identity formation in medical education for humanistic, resilient physicians: Pedagogic strategies for bridging theory to practice. Acad Med.

[38] Kabat-Zinn J (1990). Full Catastrophe Living: Using the Wisdom of Your Body and Mind to Face Stress, Pain, and Illness.

[39] Epstein-Lubow G, McBee L, Darling E, Armey M, Miller IW (2011). A pilot investigation of mindfulness-based stress reduction for caregivers of frail elderly. Mindfulness.

[40] Foureur M, Besley K, Burton G, Yu N, Crisp J (2013). Enhancing the resilience of nurses and midwives: Pilot of a mindfulness-based program for increased health, sense of coherence and decreased depression, anxiety and stress. Contemp Nurse.

[41] Noone SJ, Hastings RP (2010). Using acceptance and mindfulness-based workshops with support staff caring for adults with intellectual disabilities. Mindfulness.

[42] Shapiro SL, Warren Brown K, Biegel GM (2007). Teaching self-care to caregivers: Effects of mindfulness-based stress reduction on the mental health of therapists in training. Train Educ Prof Psychol.

[43] Gockel A, Burton D, James S, Bryer E (2013). Introducing mindfulness as a self-care and clinical training strategy for beginning social work students. Mindfulness.

[44] Sauer-Zavala SE, Walsh EC, Eisenlohr-Moul TA, Lykins ELB (2013). Comparing mindfulness-based intervention strategies: Differential effects of sitting meditation, body scan, and mindful yoga. Mindfulness.

[45] Rosenzweig S, Reibel DK, Greeson JM, Brainard GC, Hojat M (2003). Mindfulness-based stress reduction lowers psychological distress in medical students. Teach Learn Med.

[46] Warnecke E, Quinn S, Ogden K, Towle N, Nelson MR (2011). A randomised controlled trial of the effects of mindfulness practice on medical student stress levels. Med Educ.

[47] Shiralkar MT, Harris TB, Eddins-Folensbee FF, Coverdale JH (2013). A systematic review of stress-management programs for medical students. Acad Psychiatry.

[48] Paul G, Elam B, Berhulst SJ (2007). A longitudinal study of students' perceptions of using deep breathing meditation to reduce testing stresses. Teach Learn Med.

[49] Dobkin PL, Hutchinson TA (2013). Teaching mindfulness in medical school: where are we now and where are we going?. Med Educ.

[50] Escuriex BF, Labbe EE (2011). Health care providers' mindfulness and treatment outcomes: A critical review of te research literature. Mindfulness.

[51] Jensen CG, Vangkilde S, Frokjaer V, Hasselbalch SG (2012). Mindfulness training affects attention - or is it attentional effort?. J Exp Psychol Gen.

[52] Semple RJ (2010). Does mindfulness meditation enhance attention? A randomized controlled trial. Mindfulness.

[53] Ramsburg JT, Youmans RJ (2014). Meditation in the higher-education classroom: Meditation training improves student knowledge retention during lectures. Mindfulness.

[54] Sears SR, Kraus S, Carlough K, Treat E (2011). Perceived benefits and doubts of participants in a weekly meditation study. Mindfulness.

[55] Saunders PA, Tractenberg RE, Chaterji R, Amri H, Harazduk N, Gordon JS (2007). Promoting self-awareness and reflection through an experiential mind-body skills course for first year medical students. Med Teach.

[56] Kabat-Zinn J (2014). Meditation is not for the faint-hearted. Mindfulness.

[57] Ireland MJ (2013). Meditation and psychological health: Modeling theoretically derived predictors, processes, and outcomes. Mindfulness.

[58] Carmody J, Baer RA (2009). How long does a mindfulness-based stress reduction program need to be? A review of class contact hours and effect sizes for psychological distress. J Clin Psychol.

[59] Hassed C, de Lisle S, Sullivan G, Pier C. Enhancing the health of medical students: outcomes of an integrated mindfulness and lifestyle program. Adv Health Scie Educ Theory Pract. 2009; doi:10.1007/s10459-008-9125-3 10.1007/s10459-008-9125-3 18516694

[60] Braun V, Clarke V (2006). Using thematic analysis in psychology. Qual Res Psychol.

