# Supplementary material for: Mindfulness based stress reduction for medical students: optimising student satisfaction and engagement
Source: BMC Med Educ. 2016 Aug 18;16:209. doi: 10.1186/s12909-016-0728-8 (PMC4989331; doi:10.1186/s12909-016-0728-8)
Supplement: Additional file 1: Figure S1. — Qualitative Feedback Questionnaire. Open ended feedback questionnaire. (DOCX 69 kb) [file 12909_2016_728_MOESM1_ESM.docx]

**Additional file 1: Figure S1: Qualitative Feedback Questionnaire**

Human Doctor Programme

Mindfulness Based Stress Reduction Module

Course Evaluation

Group Trainers name _____________________________

No. of sessions I attended _________________________

1. **Satisfaction with course content to date**

1 2 3 4 5

Very dissatisfied Adequate Extremely

satisfied

**2. Satisfaction with group trainer to date**

1 2 3 4 5

Very dissatisfied Adequate Extremely

satisfied

**3. Learning outcomes to date**

1 2 3 4 5

Very dissatisfied Adequate Extremely

satisfied

**4. Overall level of satisfaction with the course to date**

1 2 3 4 5

Very dissatisfied Adequate Extremely satisfied

**5. Would I recommend this course to future medical students**

1 2 3 4 5

Would definitely Would definitely not recommend recommend

**6. Further comments / suggestions / feedback**

___________________________________________________________________________________________________________________________________________________________________________________________________________________________________________________________________________________________________________
