# Supplementary material for: Mindfulness based stress reduction for medical students: optimising student satisfaction and engagement
Source: BMC Med Educ. 2016 Aug 18;16:209. doi: 10.1186/s12909-016-0728-8 (PMC4989331; doi:10.1186/s12909-016-0728-8)
Supplement: Additional file 2: Practice Points. — List of practice points relevant to the study. (DOCX 51 kb) [file 12909_2016_728_MOESM2_ESM.docx]

**Practice points:**

- Medical students and medical practitioners learn and work in a very demanding environment which means they are at an increased risk of a number of personal and psychological problems.
- Mindfulness meditation is a self-care strategy and can be adapted to the individual and their experience.
- Satisfaction with such courses may be further ameliorated by creating real life clinical placement experiences so students appreciate the true role of stress reduction measures.
- Careful consideration needs to be given with regard to achieving a balance between providing students with choices on the one hand and embedding the essential requirement of learning appropriate self-care for medical students on the other.
